# Microperimetric analysis of diabetic macular edema after navigated direct photocoagulation with short-pulse laser for microaneurysms

**DOI:** 10.1186/s40942-023-00447-y

**Published:** 2023-03-02

**Authors:** Yasuko Ikegami, Tomoyasu Shiraya, Fumiyuki Araki, Takashi Ueta, Taku Toyama, Tomohiko Yanagita, Jiro Numaga, Nobuyuki Shoji, Satoshi Kato

**Affiliations:** 1 Department of Ophthalmology, University of Kitasato School of Medicine, 1-15-1 Kitasato, Minami-Ku, Sagamihara, Kanagawa Japan; 2 Department of Ophthalmology, Graduate School of Medicine, University of Tokyo, Tokyo, Japan; 3 Department of Ophthalmology, Tokyo Metropolitan Geriatric Hospital, Tokyo, Japan

**Keywords:** Diabetic macula edema, Microaneurysms, Microperimetry, Focal laser photocoagulation, Navigated laser

## Abstract

**Background:**

Focal laser photocoagulation is an important treatment option for diabetic macular edema (DME). This study aimed to examine the retinal sensitivity (RS) and morphological changes at the coagulated site after direct photocoagulation of microaneurysms (MAs) in patients with DME using a navigated laser photocoagulator with a short-pulse duration of 30 ms.

**Methods:**

Images of early-phase fluorescein angiography were merged with images from the optical coherence tomography (OCT) map with 9 Early Treatment Diabetic Retinopathy Study grid circles, and MAs inside the edema area were selected for direct photocoagulation. The best-corrected visual acuity (BCVA), parameters of the OCT map including central retinal thickness and retinal thickness in edema range, central RS, and RS in the edema area were assessed at 1 and 3 months after the laser treatment. The RS points that overlapped with the laser spots were identified by merging the Navilas’ digital treatment reports and the microperimetry images.

**Results:**

Seventeen eyes from 14 patients were studied. The mean retinal thickness in the edema range decreased at 3 months compared with pretreatment (P = 0.042), but the BCVA, central retinal thickness, central RS, and RS in the edema area remained unchanged. Overall, 32 of 400 sensitivity points overlapped with the laser-coagulated spots. The mean RS at these spots were 22.4 ± 5.3 dB at 1 month and 22.5 ± 4.8 dB at 3 months, with no significant change from the baseline of 22.7 ± 3.5 dB.

**Conclusions:**

Retinal thickness improved in the coagulated edema area without a decrease in RS after direct photocoagulation of MAs with a short 30-ms pulse using Navilas. This promising therapeutic strategy for DME is effective and minimally invasive.

## Background

Diabetic macular edema (DME) is the main cause of visual impairment in patients with diabetes. Although the pathophysiology of DME is multifactorial and complex, microaneurysms (MAs) contribute to DME and have been targeted for photocoagulation treatment [1]. First-line therapy for DME is anti-vascular endothelial growth factor (VEGF) agents, based on large clinical trials [2–4]. However, anti-VEGF therapy requires repeated and frequent injections and incurs a significant financial burden. Furthermore, up to 50% of eyes treated with monthly anti-VEGF therapy have persistent DME and require alternative treatment [5–7].

Before the advent of anti-VEGF agents, laser photocoagulation was the standard treatment for DME based on the Early Treatment Diabetic Retinopathy Study (ETDRS) report of 1985 [8]. Direct photocoagulation for MAs (MAPC) continues to play an important role in DME treatment related to leaking MAs and focally grouped MAs [2, 7, 9]. The MAPC procedure directly closes leaking MAs and, subsequently, reduces macular thickness and macular edema [9, 10]. Before a patterned scanning laser with a short-pulse duration (20–30 ms) was introduced in 2006 [11], the laser pulse duration of conventional MAPC was 100 ms [12–14]. Conventional laser parameters are associated with laser scar expansion and partial destruction of the retinal pigment epithelium (RPE) and photoreceptor cells over time [15, 16]. Notably, the patterned scanning laser has become popular as a less invasive laser therapy [11]. In addition, because tissue invasion depends on the time of laser exposure, the short-pulse laser method causes less thermal diffusion [16, 17] as well as lowers the risk of damage to the outer retina and the lesion size [18–20]. Therefore, MAPC using short-pulse duration has several advantages for the treatment of the delicate macular area.

Retinal sensitivity (RS), evaluated via microperimetry, can quantify the retinal function more closely than visual acuity, which is commonly used. The sensitivity data obtained with microperimetry provides more detailed information about the larger macular area. Local RS decreases in response to increased retinal thickness or morphologic disruption at the photoreceptor layer [21–24]. Microperimetry may be an appropriate tool to measure these functional effects. Previous studies have evaluated the impact of conventional laser treatment for DME on RS [12, 25, 26]. Conventional macular laser decreases the perimetric sensitivity within the central 10° retina [27] or mean RS in the central 12° retina [26]. Furthermore, RS, assessed using a scanning laser ophthalmoscope, decreases markedly at the site of the laser scar 3 months after laser treatment [12].

The recently developed Navilas laser system (OD-OS, Teltow, Germany) allows physicians to accurately coagulate MAs in planned locations with an automatic eye-tracking laser delivery system [28]. The treatment locations are planned by merging imported external images, including fundus photography, optical coherence tomography (OCT), and fluorescein angiography (FA). The Navilas system can also provide a digital treatment report that exhibits the actual coagulated spots.

As mentioned above, RS changes after macular laser treatment for DME have been reported, but only the mean sensitivity of a specific area, including the non-laser area, was evaluated, not the RS of the laser-coagulated spot. By merging the Navilas’ digital treatment report and microperimetry image, the RS of the actual laser-coagulated lesion can be examined. In this study, we performed MAPC for DME with a short 30-ms pulse duration using Navilas and investigated the RS and morphological changes of the laser-coagulated lesion.

## Methods

### Patients and Study design

This prospective interventional study was approved by the Institutional Ethics Committee of the Graduate School of Medicine and the Faculty of Medicine at the University of Tokyo (#11986). Written informed consent was obtained from all patients. The procedures were conducted in accordance with the tenets of the Declaration of Helsinki.

Consecutive patients were recruited from the Department of Ophthalmology at the University of Tokyo Hospital between March 2019 and June 2020. The inclusion criteria were clinically significant DME with apparent leaking MAs and eligibility for FA. Each patient was informed about the treatment options, including anti-VEGF and steroid therapies, and the risks and benefits of laser photocoagulation. Patients who were reluctant to use anti-VEGF therapy for economic or psychological reasons, had recurrent DME despite previous treatments, and were eligible for laser photocoagulation treatment were recruited to the study. All enrolled patients underwent comprehensive ophthalmologic examinations. The best-corrected visual acuity (BCVA) was measured at baseline before laser photocoagulation and at the 1- and 3-month follow-ups. The major exclusion criteria were previous pars plana vitrectomy, intraocular surgery in the last 6 months, treatment for macular edema in the last 6 months, significant media opacities, and a diagnosis or history of any ocular disease that might influence the study results, including age-related macular degeneration, inflammatory eye disease, neurodegenerative disease, and vitreomacular traction syndrome.

### Spectral-domain (SD)-OCT: image acquisition and analysis

**Fig. 1:**
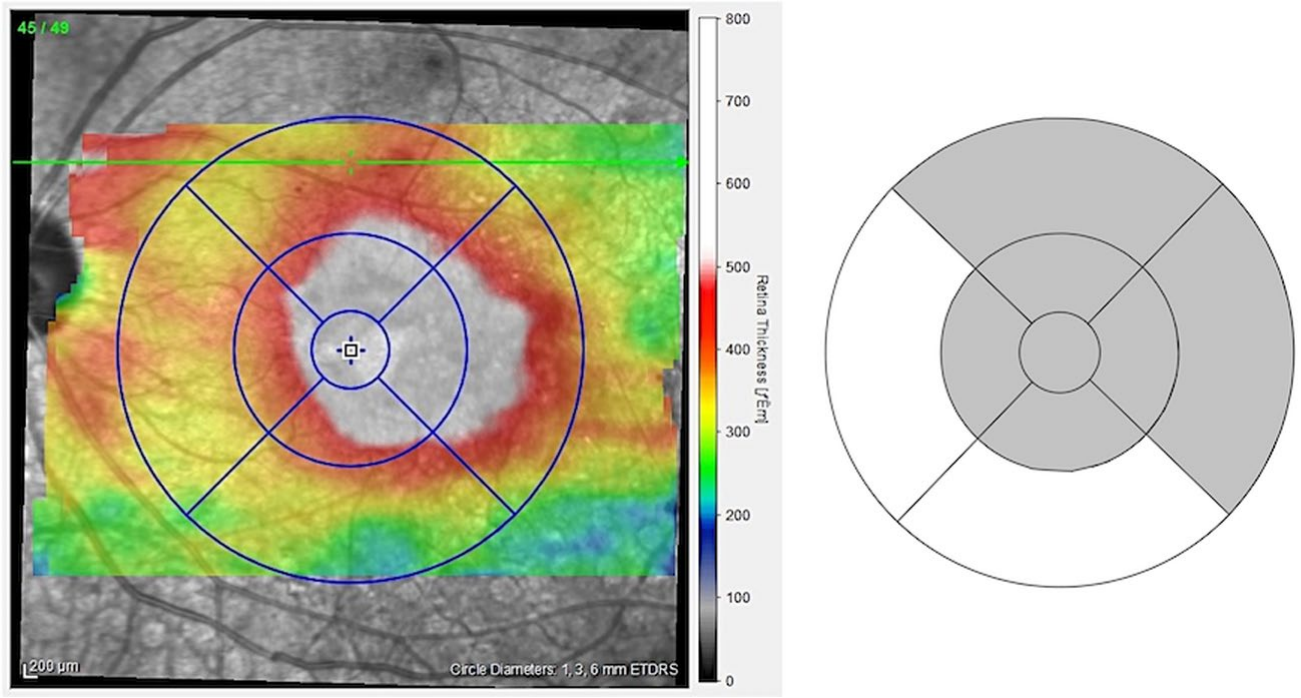
Calculation of mean retinal thickness (mRT) in the laser-treated edema area. In the optical coherence tomography retinal thickness map, the edema area is shown in red and white (> 380 µm) (Left). Using the area ratio of 9 Early Treatment Diabetic Retinopathy Study grid circles, the mRT of the laser-treated area was calculated by averaging the thicknesses of the sectors that contain more than half of the edema area (left). In the right figure, the sectors that contain more than half of the edema area are colored gray

A macular raster scan consisting of 49 B-scans and a macular thickness map of the 9 ETDRS grid circles were obtained using the built-in algorithm of the SD-OCT (Spectralis, Heidelberg Engineering Co, Heidelberg, Germany). The OCT images were acquired at baseline and 1 and 3 months after MAPC, using the follow-up function. To evaluate morphological changes, central retinal thickness (CRT) and mean retinal thickness in the treated edema area (mRT) were calculated. CRT was defined as the average thickness within the central 1-mm diameter area in the 9 ETDRS grid circles. A range with > 380 μm in the OCT map (indicated by red and white) was defined as the edema area (Fig. 1). Using the area of 9 ETDRS grid circles, the mRT was calculated by averaging the thicknesses of the sectors that contain more than half of the edema area (Fig. 1).

### Microperimetry

Microperimetry measurements were performed using the MP-3 (Nidek, Gamagori, Japan) at baseline and 1 and 3 months after MAPC treatment with the follow-up function using an implemented eye-tracking system. A 4–2 staircase strategy with a Goldmann III size stimulus was used, for a duration of 200 ms and a maximum stimulus brightness of 10,000 asb. A radial grid pattern of 40 stimulus locations covering the central area with an 18° diameter was applied. The stimulus decibel level ranged from 34 to 0 dB. The central RS was defined as the average sensitivity of the four points within the central 1-mm diameter area. The average sensitivity of the points located in the red and white areas of the OCT map was defined as the RS in the edema area.

### Navigated focal laser photocoagulation

The Navilas laser system 577 s was used with a short-pulse duration of 30 ms. The color fundus, FA (TRC-50DX, Topcon, Tokyo, Japan), and OCT images were captured by the Navilas system’s built-in software to create multimodal images. The early-phase FA was merged with the OCT thickness map, and MAs inside the edema area were selected for MAPC. Laser parameters were as follows: spot size, 50–100 µm; pulse duration, 30 ms; and laser power, 100–150 mW. The parameters were set to achieve barely visible whitening using a Volk Area Centralis^®^ contact lens (Volk Optical, Mentor, OH, USA). All laser photocoagulation procedures were performed by a retina specialist (Y.I.).

### Identification of the laser-coagulated points in the merged images of the Navilas and MP-3

**Fig. 2:**
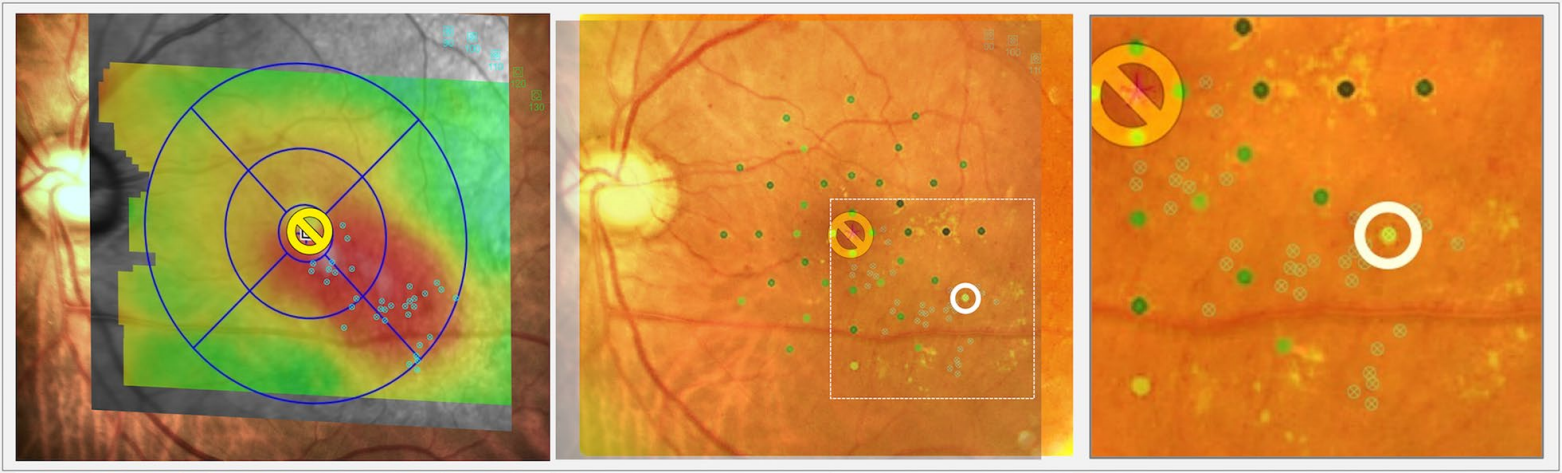
Measurement of retinal sensitivity at the laser-coagulated points. The Navilas digital treatment report showing direct photocoagulation for microaneurysms in the edema area based on a preplanned coagulation design (left). The color fundus photograph was merged with the microperimetry results and the fundus image of laser-coagulated points by Navilas. Microperimetry measurements were made with a radial grid pattern of 40 stimulus locations covering the central area with an 18° diameter. The stimulus decibel level ranged from 34 to 0 dB. Using this merged image, the areas where the laser scarring and RS point overlapped were selected for evaluation (white circle) (middle). The picture on the right shows partially enlarged images: the colored filled dots are retinal sensitivity measurement points according to microperimetry. The dot with a cross in a circle is the laser-coagulated point

Images acquired from the Navilas’ digital treatment report and RS measurements from the MP-3 were superimposed. Briefly, these two images were enlarged, made transparent using the transparency function of Microsoft PowerPoint^®^, and exactly superimposed using the corresponding retinal landmarks, including the shape and branching points of the retinal blood vessels. The merging images were carefully created by trying to merge them multiple times to ensure they overlap correctly. The overlapping points between the laser-coagulated scar and the RS points were selected, and the RS at these points was evaluated (Fig. 2).

### Statistical analysis

Data are expressed as means ± standard deviations. Normally distributed data were analyzed using the Kolmogorov–Smirnov test. Paired t-tests with Bonferroni post-hoc tests were used to compare the BCVA, CRT, mRT, central RS, RS of the edema range, and RS of the actual laser-coagulated points. P values < 0.05 were considered significant. The sample-size calculation is based on the primary hypothesis of detecting a change of 10% in RS or retinal thickness 3 months after laser therapy in treating DME with laser, with power of 80% and a confidence level of 95%. All statistical analyses were performed using EZR (Saitama Medical Center, Jichi Medical University, Saitama, Japan) [29].

## Results

Table 1 shows the baseline characteristics of the 17 eyes from 14 patients with DME. The mRT decreased from 430.1 ± 58.3 μm at baseline to 414.0 ± 52.2 μm at 3 months postoperatively (P = 0.042); however, the BCVA and CRT did not change significantly from baseline (at 3 months, P = 0.57 and P = 0.14, respectively).

**Table 1:** Baseline characteristic of study patients

Number of eyes	17
Age, years	67.2 ± 9.9
Sex, men/women	12/2
Type of diabetes, Type 1 / Type 2, n	6/11
Diabetic retinopathy stage, NPDR/PDR, n	16/1
Duration of suffering diabetes, years	12.3 ± 9.4
Duration of macular edema, years	2.9 ± 2.5
HbA1c (%)	7.6 ± 1.1
BCVA (log MAR)	0.44 ± 0.31
Central retinal thckness (µm)	458.7 ± 115.1
Central retinal sensitivity (dB)	16.9 ± 7.6
peudophakic, n	3
Panretinal photocoagulation, n	7
Prior treatment for DME, n	
Intravitreal anti-VEGF	3
Sub-Tenon’s injection of triamcinolone acetonide	9
Photocoagulation	6

Continuous parameters are presented as mean ± SD.

*SD* standard deviation, *NPDR* non proliferative diabetic retinopathy, *PDR* proliferative diabetic retinopathy, *HbA1c* glycated haemoglobin, *BCVA* best corrected visual acuity, *DME* diabetic macular edema, *VEGF* vascular endothelial growth factor

Mean central RS at postoperative months 1 and 3 were 18.3 ± 8.7 dB and 17.5 ± 8.8 dB, respectively, and did not significantly differ from the baseline of 16.9 ± 7.9 dB (P = 0.32, P = 1.0). The mean RS in the edema area at 1 and 3 months were 20.9 ± 6.4 dB and 21.1 ± 6.3 dB, respectively, and did not significantly differ from the baseline of 20.2 ± 6.3 dB (P = 0.72, P = 0.45). In the merged image of the Navilas system’s digital treatment report and the MP-3 image, 32 of the 400 sensitivity points in all 17 eyes overlapped with the laser spot. RS at these laser spots were 22.7 ± 3.5 dB at baseline, 22.4 ± 5.3 dB at 1 month, and 22.5 ± 4.8 dB at 3 months, with no significant changes during the follow-up period (P = 1.0, P = 1.0, respectively).

## Discussion

DME was treated with direct photocoagulation of the MAs using a Navilas laser system with a short 30-ms pulse duration. Changes in retinal thickness and RS were evaluated by merging the OCT image, the digital treatment report from the Navilas, and the microperimetry data image. Three months after laser therapy, the mRT improved without decreasing RS, even where the laser-treated spots overlapped.

After laser therapy using standard modified ETDRS photocoagulation, RS decreased from 12 to 52 weeks [12, 13, 26]. Furthermore, RS decreased by 13 dB or more in the retinal area overlying the laser scars, similar to the reduced RS above blood vessels (angioscotoma) or above circinate rings [12]. In contrast, in this current study, after direct photocoagulation of the MAs using the Navilas laser system with a short pulse duration of 30 ms, no reduction in RS in the coagulated edema area or in the actual coagulation spots was observed.

The lack of effects on RS may be due to the short-pulse duration of 30 ms instead of 100 ms during laser photocoagulation. The modified ETDRS protocol uses a combination of grid laser and MAPC with a longer pulse duration of 100 ms [12, 13, 26]. However, this laser parameter is associated with several disadvantages, including atrophic creep, scotoma caused by heat-induced destruction of the retina, and decreased vision [30–32]. Furthermore, in vitro experiments with cultured RPE cells demonstrate that laser photocoagulation at a power that produces barely visible laser burn not only increases the expression of heat shock protein 70, which triggers the therapeutic effects on DME, but also the expression of genes that induced cell apoptosis [33]. For these reasons, RS was reduced after laser therapy using the conventional focal/grid laser. In contrast, the advantages of short-pulse laser include reduced RPE/outer retina damage and smaller lesion size due to the reduced spread of heat [16, 18, 34, 35]. Decreased lesion size may also be attributed to the stable RS after laser treatment. Immediately after treatment, laser photocoagulated lesions exhibit a local loss of photoreceptor cells. Photoreceptors migrating from unaffected areas located at the border of the lesion fill in the damaged photoreceptor layer within 4 months [16, 20]. This morphological recovery may improve RS [16, 36]. However, photoreceptor migration to repair laser-induced damage may lead to decreased photoreceptor density in the macular area with an overall reduction in functional sensitivity [36]. The advantage of the short-pulse laser is the reduced lesion size compared with the conventional laser with a 100-ms pulse. The smaller lesion may lead to decrease in photoreceptor loss, thereby preventing the reduction in sensitivity.

Another factor contributing to the stable RS after laser therapy is the use of the Navilas system. The Navilas system, which is designed to accurately coagulate MAs at preplanned treatment locations, offers precise, safe, and effective laser treatment [28]. The accurate Navilas system can target MAs close to the macula and coagulate numerous MAs in the edematous area, which is difficult by the treatment method involving ordinary slit-lamp–based laser devices. The reported MA hit rate is 92% for Navilas compared to 72% for the standard manual laser technique [28]. The superior accuracy facilitates the coagulation of MAs at the appropriate power and in the appropriate location, preventing RS loss due to retinal damage.

Treatment of DME with the short-pulse laser method has several advantages in clinical practice. If the laser beam does not hit the MAs, scarring on the RPE will occur. However, MAPC with short-pulse duration suppresses tissue damage not only when the laser hits the MAs successfully, but also when failing to hit the targeted MAs. In addition, the number of MAs increases as DME progresses [37], and retreatment is frequently required for DME. Even during retreatment, using navigated MAPC with short-pulse duration may be less invasive.

Although the observation period after laser treatment was limited to 3 months, the expansion rate of coagulated spots after treatment with a short-pulse laser (20 ms) is significantly lower than the expansion rate of conventional lasers (200 ms) during observation periods up to 12 months postoperatively [18]. Maximum autofluorescence of laser scars is apparent 1 month after treatment and remains nearly unchanged for up to 6 months, and the morphology one month after laser therapy may indicate subsequent morphology [38, 39]. Although we followed the progress after laser treatment for only 3 months, the findings of this study are meaningful. We confirmed the reduction of mRT by MAPC with a short-pulse duration, taking advantage of using the Navilas. We believe that this laser method is safe and helpful, inducing less damage to the RPE and outer retinal layers and preventing RS decrease.

Focal/grid laser photocoagulation was once used as the standard therapy for DME, but currently anti-VEGF agents are the first-line treatment. Several clinical trials have shown the superiority of anti-VEGF treatment over laser therapy in terms of clinical efficacy, both functional (BCVA) and anatomical (retinal thickness) outcomes of the DME therapy [40–43]. However, it is known that significant numbers of anti-VEGF-resistant DMEs recur despite multiple, frequent injection, which poses a heavy economic and psychological burden to patients with DME [5–7]. Anti-VEGF-resistant DME requires alternate treatment, and laser photocoagulation is an effective alternative for the treatment of DME. A more recent laser application in the treatment of DME is subthreshold laser photocoagulation. The aforementioned studies have demonstrated the destructive aspects of conventional focal/grid laser. To overcome these disadvantages, subthreshold grid laser therapy has been proposed as an effective and minimally invasive therapy for DME [44–48]. There are now several manufacturing supplying threshold laser systems, such as Micropulse laser™ (Iridex Corp.; Quantel Medical), Endpoint Management™ (Topcon), and Microsecond Pulse (Navilas OD-OS). Although Navilas can be used for Microsecond Pulse treatment, it was not used in this study because it is a relatively new device and subthreshold laser parameters for DME need to be further clarified. Future studies are needed to determine the treatment results, including RS, with the MAPC + Microsecond Pulse (focal/grid) macular laser using Navilas.

Unfortunately, in our study, no statistically significant improvements in CRT and BCVA were observed during the follow-up period. This lack of effect may be due to the relatively low macular thickness of the treated eyes (preoperative mean CRT was 458.7 ± 115.1 μm). Furthermore, the pretreatment values were relatively good. The pretreatment RS in previous reports was 5–13 dB [12, 25, 26]; however, in our study, the mean RS within 4°, the edema range, and the direct coagulation point were 16.9 ± 7.9 dB, 20.2 ± 6.3 dB, and 22.7 ± 3.4 dB, respectively. Moreover, since anti-VEGF therapy is the first-line treatment, the number of cases for which MAPC was indicated was small, and the sample size was too small to produce significant differences in treatment effects. The other limitation of our study is that it lacked a control group. Further studies should be conducted to confirm these results.

## Conclusions

MAPC with a short 30-ms pulse using Navilas improved retinal thickness in the edema area without a decrease in RS after the procedure. These findings demonstrate the safety and efficacy of this macular laser treatment method and support the use of this method as a new therapeutic approach for DME.

## Data Availability

The datasets used during the current study are available from the corresponding author upon reasonable request.

## Abbreviations

DME: Diabetic macular edema
MA: Microaneurysm
VEGF: Vascular endothelial growth factor
MAPC: Direct photocoagulation for MAs
ETDRS: Early treatment diabetic retinopathy study
RPE: Retinal pigment epithelium;
RS: Retinal sensitivity
BCVA: Best-corrected visual acuity
OCT: Optical coherence tomography
FA: Fluorescein angiography
CRT: Central retinal thickness
RT: Retinal thickness
